# Barbed suture-assisted tension reduction and de-epithelialization for clip closure of a fecalith-associated rectal fistula

**DOI:** 10.1055/a-2946-2037

**Published:** 2026-09-22

**Authors:** Zhijun He, Yanhua Nong, Xiaotong Wang, Yunxiao Liang, Wenhua Zhang

**Affiliations:** 1 Medical Education Center 47885 Jinan UniversityGuangzhouGuangdongChina; 2 Department of Gastroenterology 477292 People's Hospital of Guangxi Zhuang Autonomous RegionNanningGuangxiChina; 3 Departments of Gastrointestinal, Hernia and Enterofistula Surgery 477292 People's Hospital of Guangxi Zhuang Autonomous RegionNanningGuangxiChina

Endoscopic approaches, including endoscopic clips, stenting, and suturing, provide minimally invasive options for gastrointestinal fistulas, although their efficacy may be limited in complex cases. 1​ ​2​

A 71-year-old woman, 12 years post–rectal cancer resection, presented with a chronic rectal fistula. Colonoscopy showed a 0.7-cm fistulous opening at the colorectal anastomosis ( Fig. 1 ). Abdominal computed tomography revealed a 2.8-cm high-density fecalith impacted within the fistula tract ( Fig. 2 ).

**Fig. 1:**
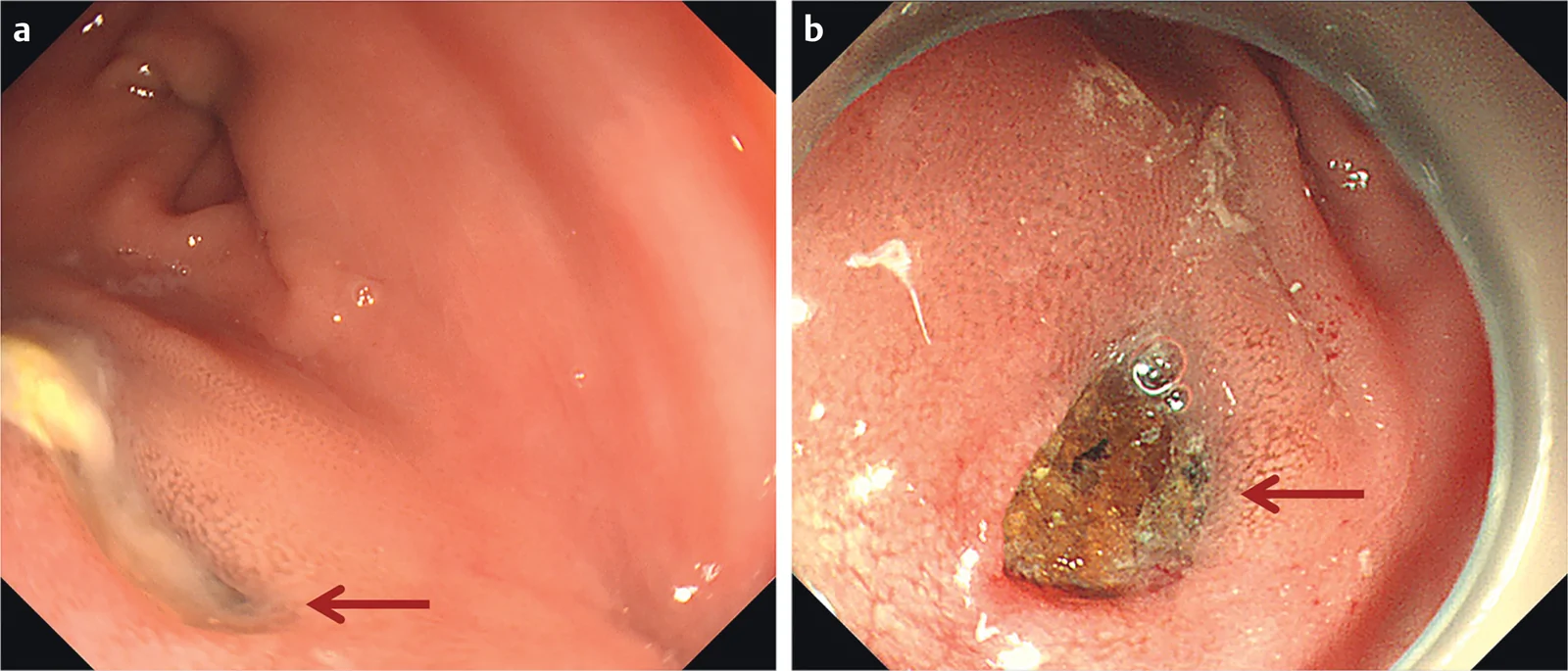
( **a** ) A rectal fistula orifice (red arrow). ( **b** ) An intraluminal fecalith (red arrow).

**Fig. 2:**
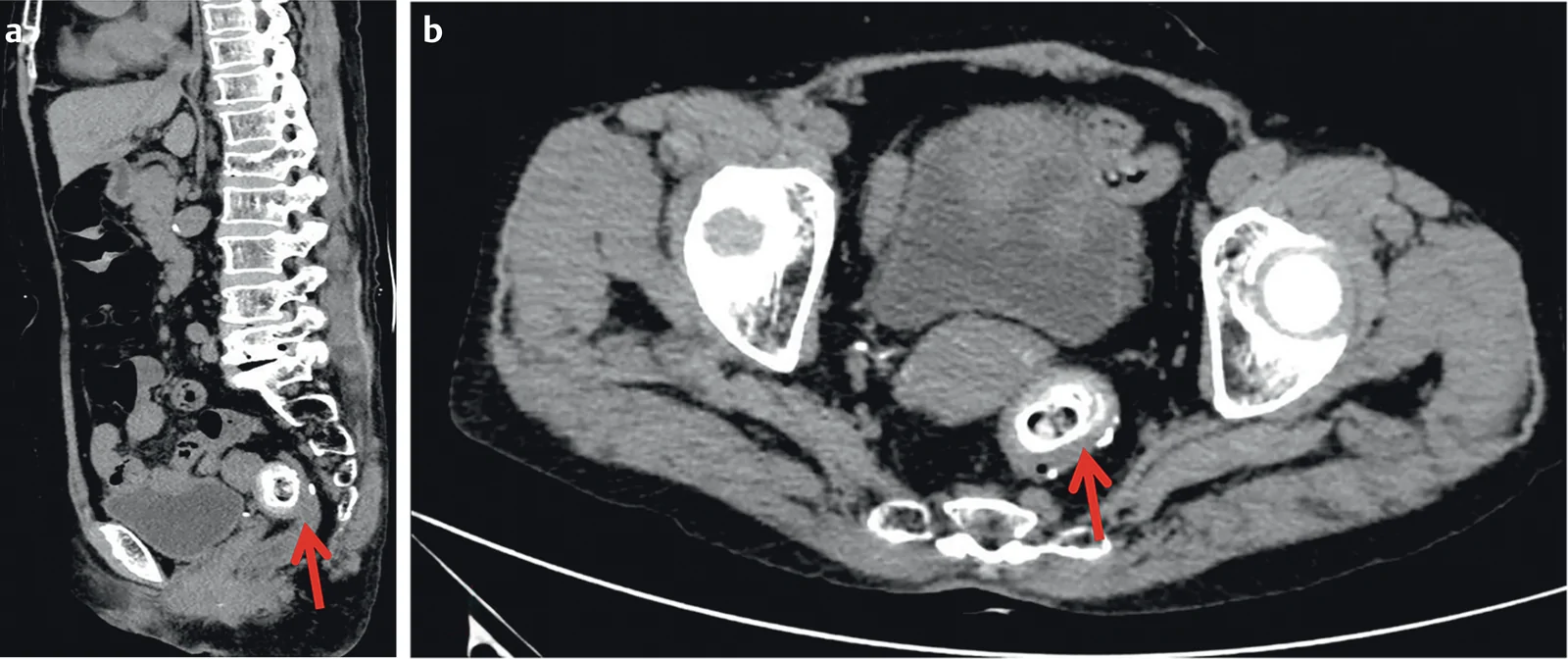
( **a** and **b** ) Abdominal CT: a 2.5-cm hyperdense perirectal fecalith (red arrow).

Endoscopic extraction followed by fistula closure was planned. After gradual dilation of the fistula tract using the gastroscope shaft, the endoscope was advanced into the cavity. The fecalith was fragmented and extracted with snares and forceps ( Fig. 3 ).

**Fig. 3:**
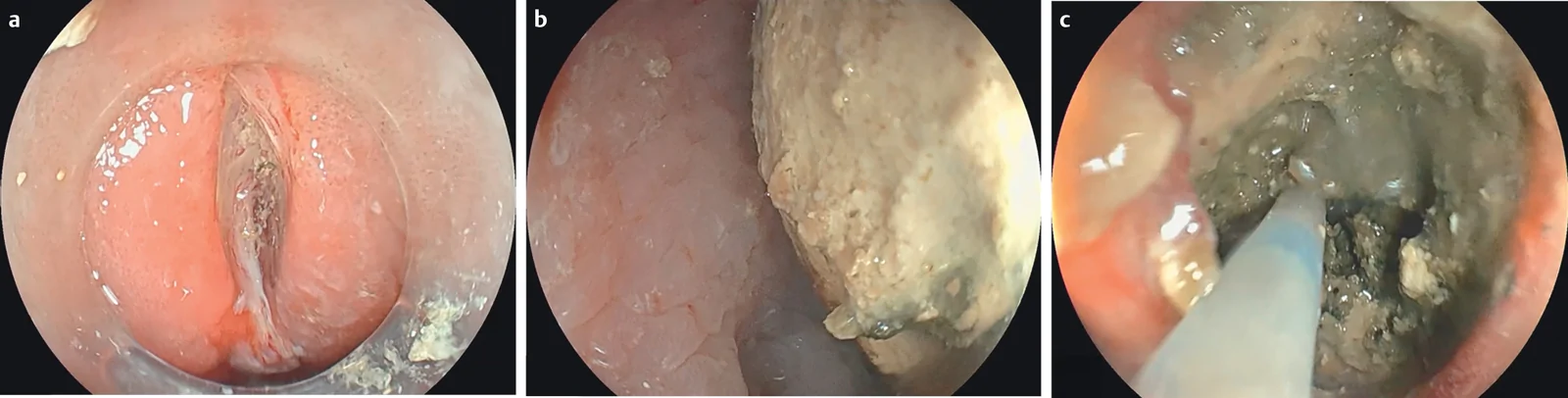
( **a** ) Fistula dilated to facilitate endoscopic entry. ( **b** ) A fecalith within the fistula. ( **c** ) Fecalith fragmentation.

Partial approximation of the fistula edges was first achieved using a transanal barbed suture technique to reduce local tension. Subsequently, circumferential electrocoagulation was applied to de-epithelialize the fistula edges. Finally, a 19 mm endoscopic clip was deployed to completely close the fistula opening ( Fig. 4 , Video 1 ). Follow-up endoscopy and contrast radiography performed 3 to 4 weeks later confirmed complete fistula healing without residual leakage ( Fig. 5 ).

**Fig. 4:**
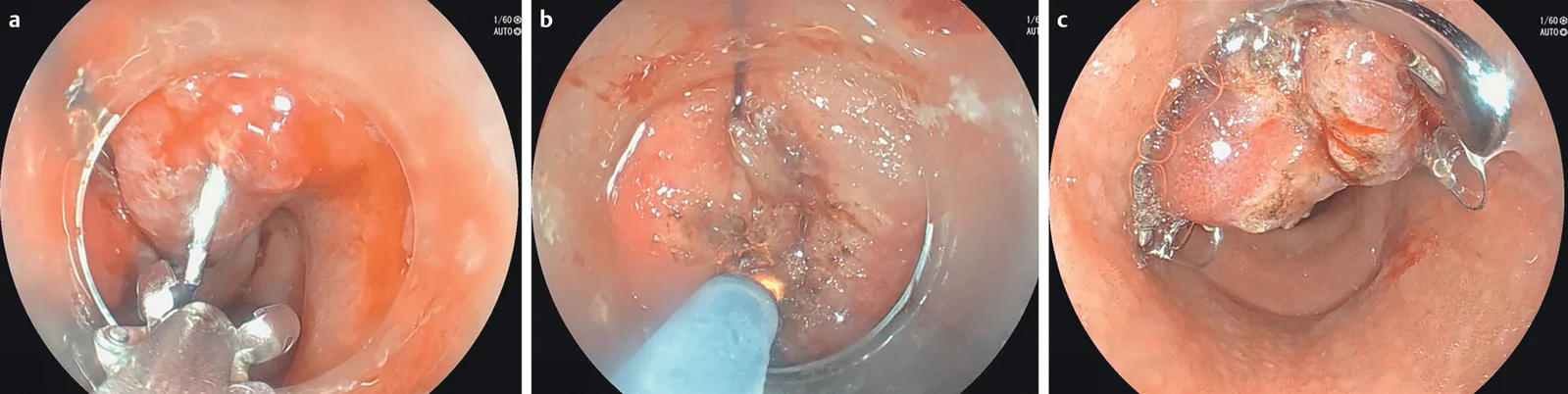
( **a** ) Barbed suture closure. ( **b** ) Circumferential electrocoagulation of fistula edges. ( **c** ) Endoscopic clip fistula closure.

**Video 1** Endoscopic management of chronic rectal fistula with fecalith extraction, barbed suture-assisted tension reduction, edge de-epithelialization, and endoscopic clip closure.


**Fig. 5:**
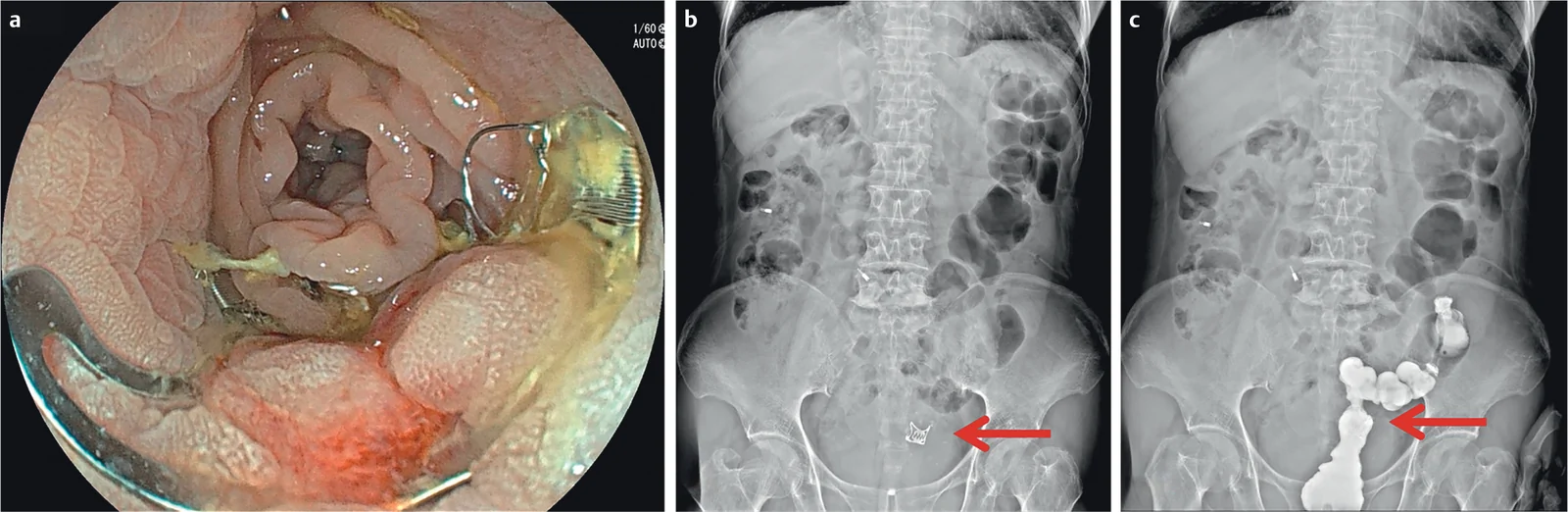
( **a** ) Three-week colonoscopy: fistula well-closed. ( **b** and **c** ) Four-week contrast radiography showing﻿: no leakage and the endoscopic clip (red arrow).

Endoscopic clip-assisted closure has demonstrated efficacy in gastrointestinal fistulas; however, success may decrease in larger or chronic fistulas because of fibrosis, epithelialization, and excessive tissue tension. 3​ Electrocoagulated edges enhance tissue engagement and positioning for the clip, and may promote fistula healing through de-epithelialization. 4​ In the present case, barbed sutures provided sustained tension reduction without knot tying and minimized suture slippage, thereby facilitating the approximation of the fistula edges before endoscopic clip deployment.

This combined minimally invasive strategy—barbed suture–assisted tension reduction, edge electrocoagulation, and endoscopic clip closure—offers a feasible rescue option for larger or chronic colorectal fistula refractory to the endoscopic clip alone; further studies are needed to assess long-term efficacy and reproducibility.
